# Lack of PRAME Expression in Cutaneous T-Cell Lymphomas

**DOI:** 10.3390/dermatopathology9010002

**Published:** 2021-12-31

**Authors:** Chau M. Bui, Sumire Kitahara, Wonwoo Shon, Tatsiana Pukhalskaya, Bruce R. Smoller

**Affiliations:** 1 Department of Pathology and Laboratory Medicine, Cedars Sinai Medical Center, Los Angeles, CA 90048, USA; sumire.kitahara@cshs.org (S.K.); wonwoo.shon@cshs.org (W.S.); 2 Department of Pathology and Laboratory Medicine, University of Rochester Medical Center, Rochester, NY 14642, USA; Tatsiana_Pukhalskaya@URMC.Rochester.edu (T.P.); Bruce_Smoller@URMC.Rochester.edu (B.R.S.)

**Keywords:** PRAME, cutaneous T-cell lymphoma

## Abstract

Cutaneous T-cell lymphomas (CTCLs) are rare tumors with no established markers that can reliably distinguish between benign and malignant lesions. Preferentially Expressed Antigen in Melanoma (PRAME) is a cancer/testis antigen that is found in many solid and hematologic malignancies. PRAME overexpression typically portends a poor prognosis and lower chemotherapeutic response. To date, no studies have established a role for PRAME in CTCL. An analysis was performed on 47 cases definitively diagnosed as CTCL: 25 cases of mycosis fungoides, 2 of Sezary syndrome, 5 of CD30+ lymphoproliferative disorder, 7 of primary cutaneous anaplastic large T-cell lymphoma, 3 of primary cutaneous CD4+ small/medium T-cell lymphoproliferative disorder, 1 of subcutaneous panniculitis-like T-cell lymphoma, and 4 of angiocentric T-cell lymphoma. PRAME immunohistochemistry was completely negative in all cases. PRAME expression was not found in any CTCL subtypes, suggesting that the pathogenesis of CTCL is not mediated by PRAME. Further study is required to identify biomarkers that might aid in the diagnosis and prognostication of CTCLs.

## 1. Introduction

Cutaneous T-cell lymphomas (CTCLs) are a rare class of tumors with an annual incidence of approximately 0.5 in 100,000 [1]. Mycosis fungoides (MF) is the most common form of CTCL, accounting for between 44% and 62% of all CTCLs [2]. While most subtypes of CTCL have an indolent clinical course, there are a number of more aggressive variants that have a very poor prognosis. The diagnosis of MF and other CTCLs can be difficult and requires a combination of the clinical examination, histopathologic evaluation, immunophenotyping, and molecular analysis [3,4,5,6,7,8,9,10,11,12,13]. To date, there are no established molecular markers that can reliably be used to diagnose malignant T cells found in a suspicious skin lesion or to distinguish aggressive from indolent CTCL subtypes. Polymerase chain reaction (PCR) and next generation sequencing (NGS) can be used to identify a clone of T cells expressing an identical rearranged copy of the T-cell receptor (TCR) gene, but these tests may fail to identify early-stage lesions [3,14,15]. A method that uses immunohistochemistry (IHC) to assist diagnosis and predict prognosis would have great clinical utility, given the superior turnaround time and cost effectiveness with IHC as compared to molecular testing.

Preferentially Expressed Antigen in Melanoma (PRAME) is a cancer/testis antigen (CTA). The CTA family comprises a number of genes whose expression is typically restricted to only male germ cells. These antigens are abnormally re-expressed in a variety of solid and hematologic malignancies [16,17,18,19,20,21,22,23,24,25,26,27,28,29,30]. PRAME overexpression is believed to prevent cell cycle arrest and apoptosis by inhibiting the retinoic acid signaling pathway. However, the molecular functions of PRAME in tumor cells remain largely unknown and may differ among different tissue types. Many studies have reported that PRAME is a helpful marker to distinguish between malignant melanoma and benign nevi. PRAME overexpression generally portends a poor prognosis with shorter overall survival and progression-free survival [22,23,24,25,28,31,32,33,34]. Due to its highly restricted pattern of expression, PRAME has emerged as an attractive target for cancer immunotherapy, in which cytotoxic T lymphocytes are developed to selectively target and eliminate PRAME-positive cancer cells [21,35,36,37,38].

To date, no studies have established a role for PRAME in the diagnosis and prognosis of CTCLs. We hypothesized that PRAME might be a useful marker to assist in the diagnosis of CTCLs and to differentiate between indolent and aggressive lesions.

## 2. Materials and Methods

This is a retrospective analysis utilizing pre-existing tissue from patients with a definitive diagnosis of CTCL by biopsy. For MF in particular, tumors at either the patch stage or more advanced stages (plaque and tumoral) were included. Patients younger than 18 years old or with concomitant malignancy of the skin at other sites were excluded. Specimens were obtained from the surgical pathology archive of a tertiary medical center over a 10-year span. Unstained recuts were obtained from the original formalin-fixed paraffin-embedded tissue (FFPET) blocks. Hematoxylin and eosin (H&E) staining and immunohistochemistry for PRAME (EPR20330 rabbit monoclonal antibody) with red chromogen were performed on recut slides.

All cases were reviewed by two independent investigators to confirm the diagnoses and evaluate PRAME expression. The following parameters were evaluated: percentage of PRAME expression, nuclear/cytoplasmic expression, and intensity and extent of staining. Melanoma and sebaceous glands were used as external and internal positive controls, respectively. The results were tabulated and compared utilizing statistical software in Excel and SPSS. A chi-squared test was used to compare differences in PRAME expression between malignant T cells in CTCL and adjacent normal sebaceous glands or melanoma tissue as a control group.

## 3. Results

The study consisted of 47 CTCLs from patients aged 26 to 91 years with an average age of 59.2, with 31 male (66.0%) and 16 female (34.0%) patients. There were 28 white (59.6%), 8 black (17.0%), 2 Asian (4.3%), 2 other non-Hispanic (4.3%), and 7 patients of unknown ethnicity (14.9%).

**Figure 1:**
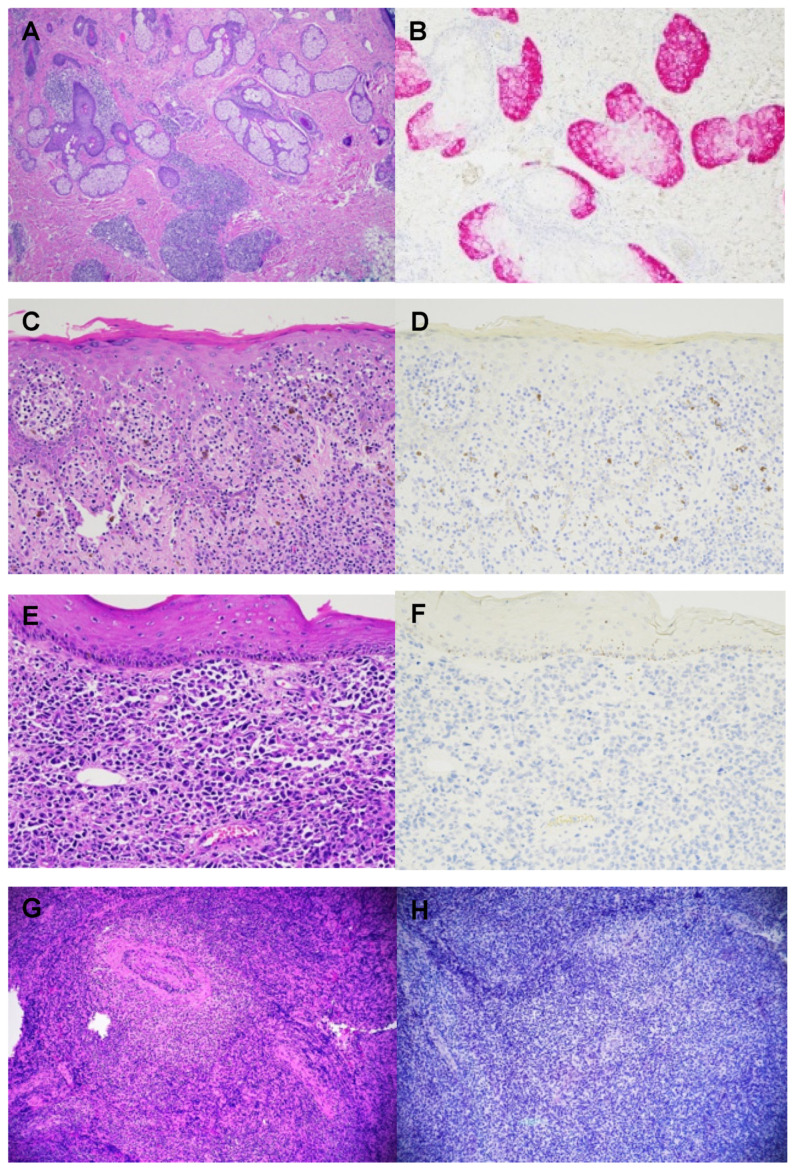
(**A**,**B**). H&E and PRAME stain of primary cutaneous small/medium CD4+ T-cell lymphoproliferative disorder with normal sebaceous glands (internal positive control), (**C**,**D**). H&E and PRAME stain of MF, (**E**,**F**). H&E and PRAME stain of primary cutaneous anaplastic large cell lymphoma, (**G**,**H**). H&E and PRAME stain of angiocentric T-cell lymphoma.

The sample included 25 cases of mycosis fungoides (53.2%), 2 of Sezary Syndrome (4.3%), 5 of CD30+ lymphoproliferative disorder (10.6%), 7 of primary cutaneous anaplastic large T-cell lymphoma (14.9%), 3 of primary cutaneous CD4+ small/medium T-cell lymphoproliferative disorder (6.4%), 4 of angiocentric T-cell lymphoma (also known as extranodal NK/T-cell lymphoma, nasal type) (8.5%), and 1 of subcutaneous panniculitis-like T-cell lymphoma (2.1%). Of the 25 MFs, all stages (patch, plaque, and tumoral) were represented, including 3 cases with large cell transformation (12.0%), 2 with the CD8 immunophenotype (8.0%), and 1 with blast cell transformation (4.0%). PRAME immunohistochemical staining was strongly and diffusely positive in both the external positive control—melanoma tissue—and the internal positive control—benign sebaceous glands, whereas the stain was completely negative in all malignant T cells (Table 1, Figure 1). The background cells, including reactive lymphocytes, were also negative for PRAME in all cases.

## 4. Discussion

The function of PRAME in normal and tumor cells is not completely understood, although a role in the regulation of retinoic acid signaling and immune evasion has been proposed. The regulation of PRAME gene expression is also poorly understood, and thus the molecular basis of its expression in malignancies is largely unknown. While PRAME is absent or expressed at very low levels in most normal tissues, high levels of PRAME mRNAs are encountered in certain types of malignant cells. PRAME expression has been described in a variety of hematologic malignancies, including both acute and chronic myeloid and lymphocytic leukemias, hairy cell leukemia, Hodgkin’s lymphoma, diffuse large B-cell lymphoma, mantle cell lymphoma, and multiple myeloma. PRAME overexpression in these tumors generally portends a poor prognosis with shorter overall survival and progression-free survival and lower chemotherapeutic response [25,26,27,28,29,30,31,32,33,34]. Some studies have described a favorable prognosis with PRAME overexpression in both acute myeloid and lymphoblastic leukemia in pediatric patients as well as acute myeloid leukemia in adults [39,40,41,42]. There have been no studies of PRAME expression in either cutaneous or non-cutaneous T-cell lymphoma.

While most cases of CTCL have an indolent clinical course, some subtypes have a very aggressive course. For instance, MF is typically a slow-growing tumor, but a small subset of cases can progress quickly to large cell or blast cell transformation, which is associated with worse prognosis. In 2018, Masson D et al. found that in CTCLs—particularly MF—a tumor clone frequency >25% (measured by high-throughput sequencing of the TCRB gene) is an independent predictor of early disease progression and death [43]. However, to date, there are no established molecular markers that can be used to reliably predict malignant potential in T cell neoplasms. We hypothesized that PRAME might have value as a molecular “fingerprint” to help with this distinction given its correlation with disease progression in other hematologic malignancies. However, PRAME expression was not found in any of the CTCL lymphoma subtypes assessed in this study, including both indolent (MF, CD30+ lymphoproliferative disorder, primary cutaneous anaplastic large T-cell lymphoma, primary cutaneous CD4+ small/medium T-cell lymphoproliferative disorder, and subcutaneous panniculitis-like T-cell lymphoma) as well as aggressive (leukemic MF, Sezary Syndrome, and angiocentric T-cell lymphoma) subtypes.

This study had several limitations that should be acknowledged. Firstly, only one PRAME clone was used in the study; the results may have differed if another clone were used due to variable immunohistochemical staining patterns among different PRAME clones. Secondly, we did not stain all subtypes of CTCLs, with the notable omission of primary cutaneous gamma-delta T-cell lymphoma, primary cutaneous acral CD8+ T-cell lymphoma, and primary cutaneous CD8+ aggressive epidermotropic cytotoxic T-cell lymphoma due to the extreme rarity of these entities. As such, the results of this study cannot be generalized to all subtypes of CTCL.

## 5. Conclusions

In summary, our study suggests that the pathogenesis of CTCL is not mediated by PRAME. Further study is required to identify additional biomarkers that might aid in the diagnosis and prognostication of CTCL.

## Figures and Tables

**Table 1:** PRAME immnohistochemistry in CTCLs.

CTCLs	Number of Cases (%)	PRAME Immunohistochemistry
MF	25 (53.2)	Negative
MF with CD8 immunophenotype	2 (8.0)	Negative
MF with large cell transformation	3 (12.0)	Negative
MF with blast cell transformation	1 (4.0)	Negative
Sezary Syndrome	2 (4.3)	Negative
CD30+ lymphoproliferative disorder	5 (10.6)	Negative
Primary cutaneous anaplastic large T-cell lymphoma	7 (14.9)	Negative
Primary cutaneous CD4+ small/medium T-cell Lymphoproliferative disorder	3 (6.4)	Negative
Angiocentric T-cell lymphoma	4 (8.5)	Negative
Subcutaneous panniculitis-like T-cell lymphoma	1 (2.1)	Negative

## Data Availability

The data supporting reported results can be found at https://pubmed.ncbi.nlm.nih.gov (accessed on 28 December 2021).
